# Exploration of Catalytic Selectivity for Aminotransferase (BtrR) Based on Multiple Molecular Dynamics Simulations

**DOI:** 10.3390/ijms20051188

**Published:** 2019-03-08

**Authors:** Ye Liu, Youzhong Wan, Jingxuan Zhu, Muxin Li, Zhengfei Yu, Jiarui Han, Zuoming Zhang, Weiwei Han

**Affiliations:** Key Laboratory for Molecular Enzymology and Engineering of the Ministry of Education, National Engineering Laboratory of AIDS Vaccine, College of Life Science, Jilin University, Changchun 130023, China; lye16@mails.jlu.edu.cn (Y.L.); wanyouzhong@jlu.edu.cn (Y.W.); zhujx15@mails.jlu.edu.cn (J.Z.); limx1315@mails.jlu.edu.cn (M.L.); yuzf16@mails.jlu.edu.cn (Z.Y.); jrhan17@mails.jlu.edu.cn (J.H.); zmzhang@jlu.edu.cn (Z.Z.)

**Keywords:** aminotransferase from *Bacillus circulans* BtrR, molecular dynamics simulation, principal component analysis, steered molecular dynamics simulation, stereoselective mechanism

## Abstract

The aminotransferase from *Bacillus circulans* (BtrR), which is involved in the biosynthesis of butirosin, catalyzes the pyridoxal phosphate (PLP)-dependent transamination reaction to convert valienone to β-valienamine (a new β-glycosidase inhibitor for the treatment of lysosomal storage diseases) with an optical purity enantiomeric excess value. To explore the stereoselective mechanism of valienamine generated by BtrR, multiple molecular dynamics (MD) simulations were performed for the BtrR/PLP/valienamine and BtrR/PLP/β-valienamine complexes. The theoretical results showed that β-valienamine could make BtrR more stable and dense than valienamine. β-valienamine could increase the hydrogen bond probability and decrease the binding free energy between coenzyme PLP and BtrR by regulating the protein structure of BtrR, which was conducive to the catalytic reaction. β-valienamine maintained the formation of cation-p interactions between basic and aromatic amino acids in BtrR, thus enhancing its stability and catalytic activity. In addition, CAVER 3.0 analysis revealed that β-valienamine could make the tunnel of BtrR wider and straight, which was propitious to the removal of products from BtrR. Steered MD simulation results showed that valienamine interacted with more residues in the tunnel during dissociation compared with β-valienamine, resulting in the need for a stronger force to be acquired from BtrR. Taken together, BtrR was more inclined to catalyze the substrates to form β-valienamine, either from the point of view of the catalytic reaction or product removal.

## 1. Introduction

Valienamine is an unsaturated cyclic alcohol pseudo-aminosaccharide with side chains and has been developed as a novel glucosidase inhibitor because of its similar chemical structure with d-glucose [1]. It can control blood glucose elevation, and various diseases caused by hyperglycemia, such as diabetes. In addition, it can effectively treat sclerosis, obesity, diabetes, and hyperlipidemia [2,3]. According to the specificity of chiral carbon atoms, valienamine has two isomers in its natural condition: valienamine and β-valienamine. The derivative (*N*-octyl-β-valienamine) of β-valienamine is an effective therapeutic agent for lysosomal storage diseases caused by the disorder of β-glycosidase [4,5,6]. Since 1980s, a number of researchers have investigated the chemical synthetic routes of *N*-octyl-β-valienamine [7,8,9,10,11,12,13]. Considering the insufficient stereospecificity of the chemical catalysts, multiple chiral carbon makes the biosynthesis of β-valienamine difficult. Moreover, harsh reactions, complex synthesis steps, and chemical pollution increase the difficulties in synthesis and economic costs. Until now, biosynthesis remains as a powerful method for the synthesis of β-valienamine. Therefore, developing a biosynthetic pathway for generating β-valienamine in vivo is necessary.

**Figure 1:**
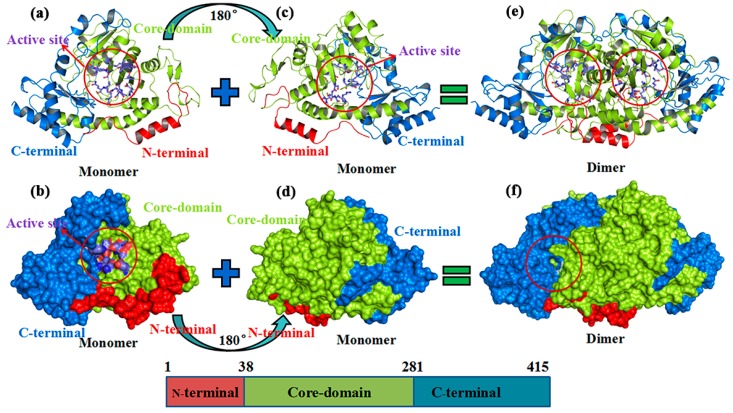
Overview of aminotransferase (PDB (protein data bank):5W71). (**a**,**b**) 3D structure of aminotransferase. The N terminal (residues 1 to 37), which contained one α-helix, is colored in red. The core domain (residues 38 to 281) is colored in deep teal. The C terminal (residues 282 to 415) is colored in forest. The active site is colored in lavender. (**c**,**d**) The aminotransferase are rotated 180. (**e**,**f**) Homologous dimer with the active site located between two monomers.

In 2016, Cui et al. [14] used a heterogeneous aminotransferase (BtrR) from *Bacillus circulans* [15,16], which can catalyze valienone to β-valienamine and maintain an optical purity of >99.9%, to generate β-valienamine in a validamycin producer named *Streptomyces hygroscopicus 5008*. Figure 1a–f shows that BtrR is composed of two homologous monomers. Each monomer is divided into three domains: α-helical N-terminus (residue 1–38), a central αβα sandwich domain (residue 39–281), and an αβ C-terminal domain (residue 282–415) (Figure 1a), which consists of 12 α-helices and 16 β-strands. Two reverse symmetrical monomers form a homologous dimer (Figure 1e,f). The active sites are located between subunits A and B. BtrR belongs to the fold type I or aspartate aminotransferase family [17,18].

The general mechanism of the aminotransferase reaction is shown in Figure S1. Pyridoxal phosphate (PLP) binds to the enzyme protein through a Schiff base formation with the ε-amino group of an active-site Lys192. The addition of a substrate amino acid to the holoenzyme causes a transaldimination, forming the external Schiff base with the substrate. One of the three bonds belonging to the α-carbon atom of the amino acid moiety is cleaved, and an anionic intermediate is formed. The α-hydrogen is initially abstracted from the amino acid moiety of the external Schiff base complex to form a planar intermediate. The transfer of hydrogen between C-2 of the substrate moiety and C-4′of the coenzyme is characteristic of enzymatic transmutation as recognized by crystallographic studies of PLP enzymes.

Although BtrR can specifically catalyze the formation of β-valienamine from valienone, the mechanism for its specific selectivity has not been studied. Previous work has failed to address the stereoselective mechanism of BtrR. At present, the following problems remain unsolved: (1) How does binding different ligands induce protein conformation? (2) Why does BtrR have such a high catalytic selectivity for β-valienamine? In this study, multiple molecular dynamics simulations were carried out to explore the conformation dynamics of two systems. This study will explore the stereospecificity for the substrate of BtrR and provide theoretical knowledge for its development. Our study will provide detailed atomistic insight into the stereospecificity for β-valienamine of BtrR.

## 2. Results and Discussion

### 2.1. β-Valienamine and Valienamine Docking to BtrR

To obtain a reasonable substrate structure, β-valienamine and valienamine were optimized according to the density functional theory at the B3LYP/6-31G* level using the Gaussian 09 software [19]. Table 1 listed the energy components (E_gap_), ionization potential (IP), and electron affinity (EA) energies of β-valienamine and valienamine. As shown in the table, the Egap of β-valienamine was lower than that of valienamine, which suggested that β-valienamine was more prone to electron transfer than valienamine. The EA and IP of β-valienamine are higher than those of valienamine, which suggested that β-valienamine could obtain electrons more easily than valienamine. This finding may be the reason why BtrR can make β-valienamine first.

**Figure 2:**
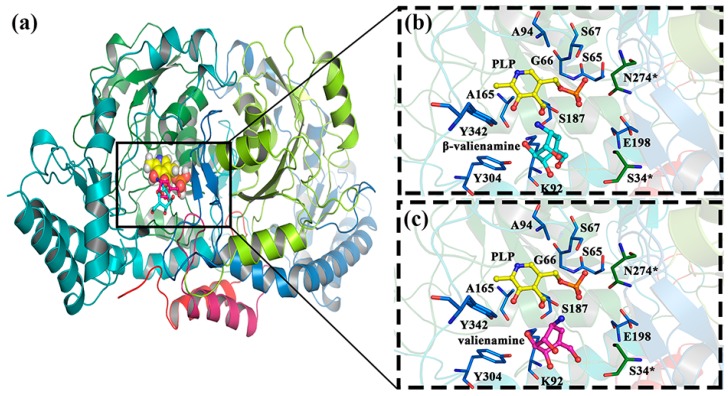
The results for docking using AutoDock Vina. (**a**) Comparison of docking results of β-valienamine and valienamine. (**b**,**c**) Close display of docking results. The residues in chain A that surround substrates and PLP are colored by marine, while they are colored by forest in chain B. β-valienamine, valienamine, and PLP are highlighted by cyan sticks, pink sticks, and yellow sticks, respectively.

By molecular docking simulation, a reliable initial model of β-valienamine, valienamine, and pyridoxal phosphate (PLP) was obtained. We docked the PLP (co-crystallized ligand) to BtrR to compare the two docking methods (AutoDock Vina and AutoDock4.2) [20]. Both docking results for AutoDock Vina and AutoDock 4.2 (Figure S2) showed that the position of the PLP, which was docked to BtrR, was similar to that in crystal (PDB ID:5W71). This finding indicated that our docking methods were reasonable. In this study, we used AutoDock 4.2 to carry out our docking study. Then, PLP/β-valienamine and PLP/valienamine were docked to BtrR. Figure 2 showed that Lys192, Tyr304, Ser187, and PLP each had a strong interaction with β-valienamine, whereas Ser187, Lyr192, and PLP exhibited interaction with valienamine. Two ligands were successfully docked to the active pocket of BtrR. It can be seen from the catalytic reaction mechanism of BtrR (Figure S1) that Lys192 and PLP play the role of catalytic residues and coenzymes, respectively. The relative positions between β-valienamine/valienamine and Lys192/PLP are shown in Figure S3. As we can see, Lys192 served as a good bridge between β-valienamine/valienamine and PLP. The relative positions of the two products were obviously different from those of Lys192. Docking results were realistic and could be used for MD simulation analyses. For clarity, two MD simulation systems were represented as BtrR/PLP/β-valienamine and BtrR/PLP/valienamine.

### 2.2. Structural Stability and Dominant Domain Motions of Two Complexes

To confirm the strong correlated conformational changes of protein regions influenced by different ligands in BtrR, correlation matrix analysis can clarify the dynamic motion that was carried out. The maps of two complexes are illustrated in Figure S4, where the large-scale and antiharmonic motions are highlighted at the diagonal of the matrix. The data for correlation was exacted using Bio3d [21], which is an R package for the comparative analysis of protein structures from trajectories. The regions that contained residues with strongly correlated motions were called positive regions and colored in blue in the map, whereas those that contained residues with anticorrelated movements were called negative regions and colored in pink. The regions at the diagonal of the map represented the positive motion of the residues with themselves, which illustrated high correlation. In normal conditions, the values of the map fluctuated between −0.1 and 0.1, suggesting that the motion of the residues was in the normal range. Figure S4 shows that valienamine induced intense centralized self-correlated motion, whereas β-valienamine could weaken this motion and produce some anticorrelated and correlated motions of BtrR. It was indicated that β-valienamine made the whole protein of BtrR fluctuate less during the 300 ns simulations. In addition, compared with other regions, the residues located at Leu100-Asn150 were accompanied by significant correlated or noncorrelated motions. The results of the correlation matrix analysis also indicated that the regions of residues C164-S167 were accompanied by significant correlated or noncorrelated motions, which undermined the modulated PLP binding.

### 2.3. β-Valienamine and Valienamine Affect the PLP Binding to BtrR

**Figure 3:**
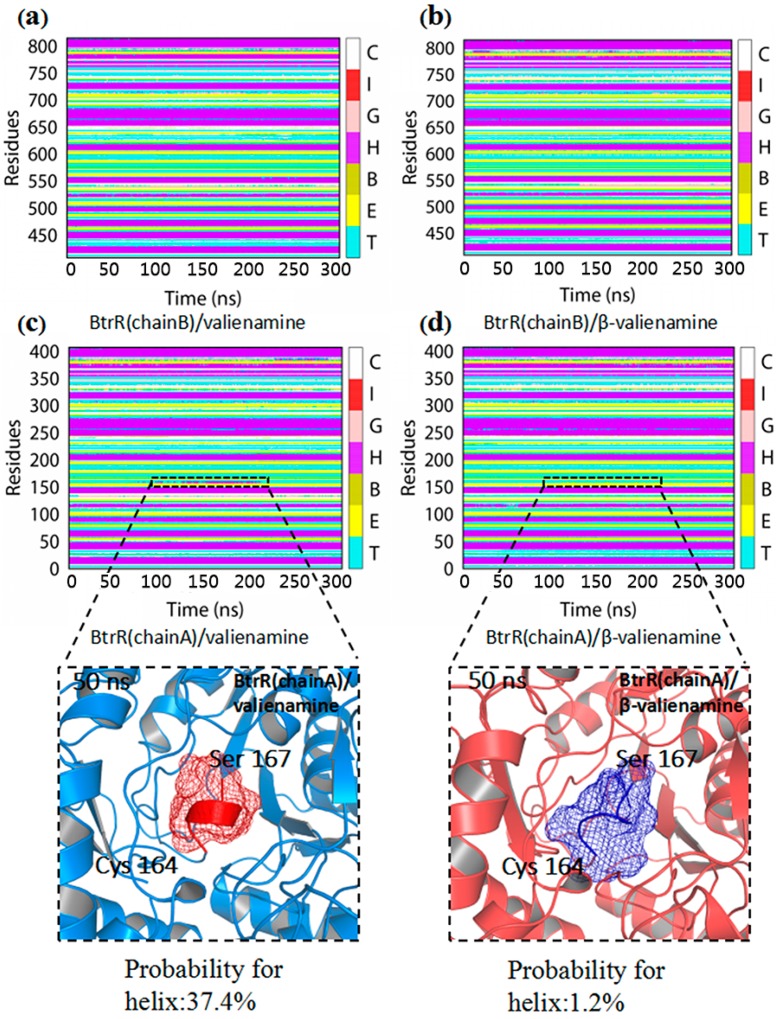
Dynamic changes of the secondary structure profile for BtrR/PLP/valienamine and BtrR/PLP/β-valienamine throughout the simulation. (**a,c**) The the secondary structure profile for BtrR/PLP/valienamine. (**b,d**) The the secondary structure profile for BtrR/PLP/β-valienamine. The colored bar represented different secondary structures as follows: coil (C), β-bugle (E), β-bridge (B), helix (G), α-helix (H), and π-helix (I).

**Figure 4:**
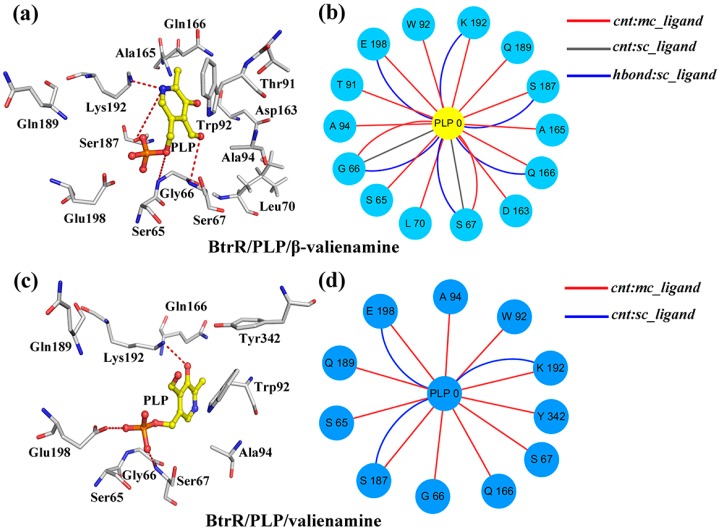
Two-dimensional view and three-dimensional view of the subnetwork of the PLP for BtrR. (**a**,**b**) The subnetwork of PLP and BtrR in the BtrR/PLP/β-valienamine complexes. (**c**,**d**) The subnetwork of PLP and BtrR in BtrR/PLP/valienamine complexes. The interaction types: interatomic contact (cnt):ligand-sc are highlighted in light gray, ligand-mc are highlighted in red, and hbond:ligand-sc is highlighted in blue.

To study the catalytic selectivity of BtrR, the secondary structure component was calculated and the results are presented in Figure 3a–c. The α-helix in residues C164-S167 was 1.2% in BtrR/PLP/β-valienamine, whereas in BtrR/PLP-/valienamine, it was approximately 37.4%. The secondary structure results illustrated that the α-helix of residues C164-S167 in BtrR/PLP/valienamine disappeared in BtrR/PLP/β-valienamine during the 300-ns MD simulations, indicating the disordered structure in the PLP binding domain, which may influence the binding of β-valienamine to BtrR. To confirm the results, the hydrogen bonds between PLP and BtrR in two complexes during MD simulation were employed and the results are shown in Table 2. There were more hydrogen bonds in BtrR and PLP in BtrR/PLP/β-valienamine than in the BtrR/PLP/valienamine complex. It is noteworthy that the probability of hydrogen bonds between PLP and Asp163/Gln166 (near the region C164-S167) increased significantly in the BtrR/PLP/β-valienamine complex. In addition, testing of the hydrogen bonds between BtrR and β-valienamine/valienamine was performed and the results are shown in Table S1. It can be seen that, compared to β-valienamine, valienamine could form more hydrogen bonds with BtrR. The distance between atoms can influence the formation of hydrogen bonds. Thus, the distance between PLP and active pocket was detected and the results are shown in Figure S5. The distance from Lys192:NZ (chain A) to PLP:O6 in the BtrR/PLP/valienamine complex was obviously larger than that in the BtrR/PLP/β-valienamine complex, and the same trend could be found between Glu198:OE2 and PLP:O5, which may be one reason for the reduction of hydrogen bonds between PLP and BtrR in the BtrR/PLP/valienamine complex. Similarly, the distance between active pocket of BtrR and β-valienamine/valienamine was also analyzed (Figure S6). It can be seen that β-valienamine exhibited a larger distance to Gly191, Lys192, and Tyr304 than valienamine, which may have decreased the hydrogen bonds between β-valienamine and BtrR. The decrease in hydrogen bond interactions may have affected the binding of BtrR. These results indicated that β-valienamine could enhance the interaction between BtrR and PLP from the point of view of protein structure changes and the probability of hydrogen bond formation. Subnetwork analysis was used to demonstrate the influence of ligands on the connection between BtrR and PLP during the simulation (Figure 4). Fourteen residues of BtrR had cnt (interatomic contact) and sc-ligand interaction with PLP in the BtrR/PLP/β-valienamine complex, which was more than that of in the BtrR/PLP/valienamine complex. Figure 4b showed that residues C164-S167 in the BtrR/PLP/β-valienamine complex maintained a strong relationship with PLP, which illustrated that β-valienamine could increase the connection between BtrR and PLP by regulating the structure of residues C164-S167.

**Figure 5:**
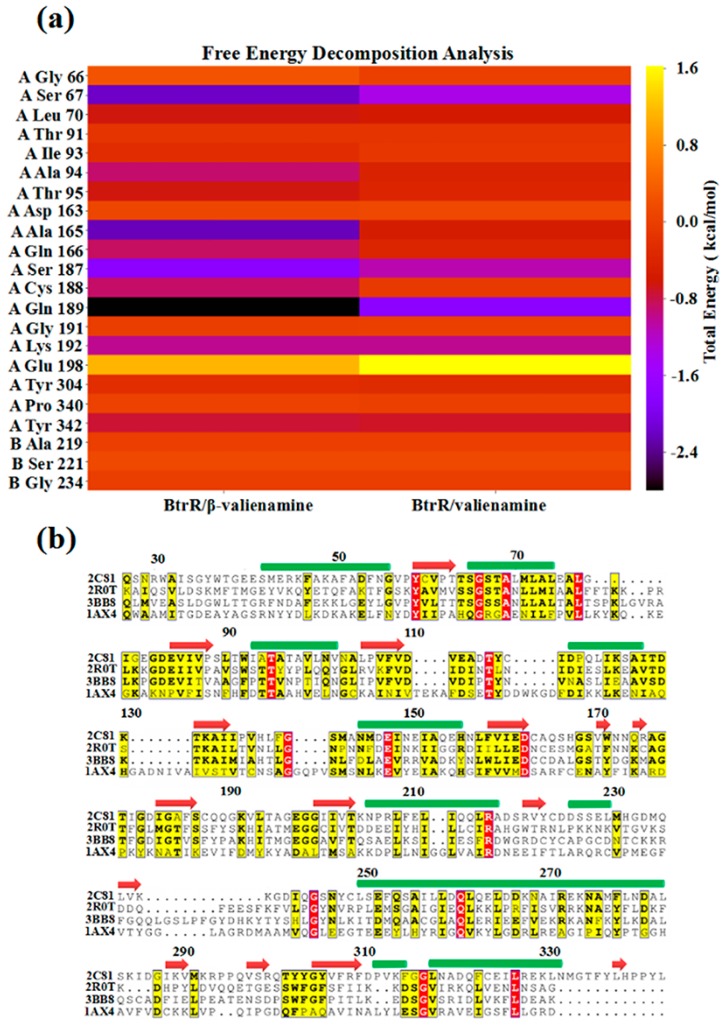
Free energy decomposition analysis for the BtrR/β-valienamine and BtrR/valienamine complexes. (**a**) The total energy for residues (calculated using MM-GBSA). (**b**) Homologous sequence correlation for PDB ID 5W71 (BtrR), 2R0T, 3BB8, and 1AX4. The residues are highlighted in red and yellow, which correspond to high homology.

It is well known that binding free energy played an important role in the analysis of the binding strength between proteins and ligands. In this study, the binding energy of BtrR and ligands was performed and the results are shown in Table S2. The binding free energies were primarily driven by polar solvation and electrostatic interactions. In addition, the vdW (van der Waals)interaction and entropy also contributed to the binding free energies. The binding energy between BtrR and PLP in BtrR/PLP/valienamine (−122.5015 kcal/mol) was higher than it in the BtrR/PLP/β-valienamine complex (−143.7635 kcal/mol), which indicated that β-valienamine made BtrR and PLP more closely integrated and provided more opportunities for interaction between PLP and BtrR. This result was consistent with that of hydrogen bond analysis (Table 2). It was interesting that compared to β-valienamine (−83.0855 kcal/mol), valienamine exhibited a lower binding free energy (−97.5237 kcal/mol). In addition, we also prepared two 30-ns simulations for both BtrR/PLP and BtrR/PLP/valienone to analyze the effect of the reactant (valienone) on the binding energies of BtrR and PLP. The molecular mechanics-generalized born surface area (MM-GBSA) results (Table S3) showed that valienone can obviously reduce the binding energy between BtrR and PLP. The per-residue binding free energies were prepared to analyze the effects of individual amino acids on PLP and BtrR binding (Figure 5a). From Figure 5a, it can be seen that the binding free energy contributions of residues in BtrR/PLP/β-valienamine (S67, A165, S187, Q189, K192) were consistently higher than that of BtrR/PLP/valienamine. Thus, the energy contribution of residues in BtrR/PLP/β-valienamine constituted the major components of total energy contribution. The residues, which were recognized as the significant contributions to the combination between PLP and BtrR, are shown in Figure S7. The results showed that β-valienamine could enhance the interaction between PLP and BtrR. Figure 5b showed that G66, S67, S187, and K192 appeared in four aminotransferases, which might be important for BtrR function.

**Figure 6:**
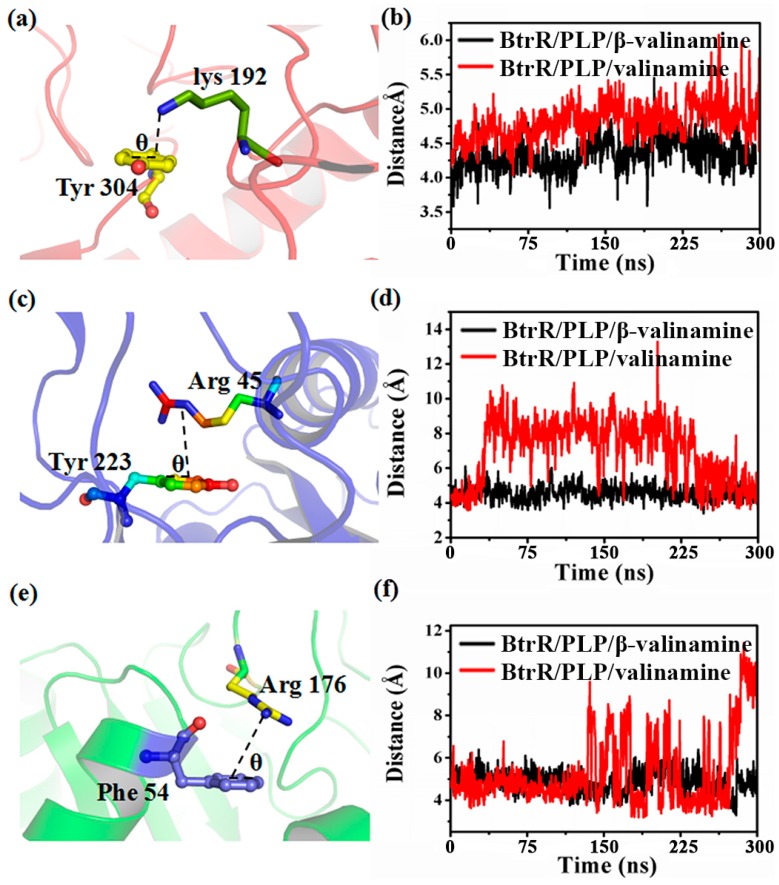
Distance between the (**a**) aromatic ring and cation of Tyr 304 and Lys192, (**c**) aromatic ring and cation of Tyr 223 and Arg45, (**e**) aromatic ring and cation of Phe45 and Arg176. Distance between (**b**) Tyr 304 and Lys192, (**d**) Tyr 223 and Arg45, (**f**) Phe45 and Arg176 was calculated from the cation to the centroid of the aromatic in chain A during the 300-ns MD of BtrR/valienamine and β-BtrR/valienamine, respectively.

Besides catalytic and isomeric centers, cation-p interaction can also affect the activity of proteins to a large extent. The cation-p interactions in two simulation systems were calculated and are shown in Table S4. Six cation-p interactions were found in each chain. Figure 6 shows the distances among Y304-R192, Y223-R45, and F54-R176 from the centroid of the aromatic ring to the cation in Figure 6b,d,f during the MD simulations. Three cation-p interactions were stable in BtrR/PLP/β-valienamine during 300-ns MD simulations, but they were not found in BtrR/PLP/valienamine. Y304 and K192 were involved in substrate binding. Y223, which was located near the active site M235 (B), was the important residue for the active site. K176 was located near the loop Q166 to V171, which modulated PLP binding. Thus, three cation-p interactions (K192 functioned as catalytic residue) disappeared, which may have affected the substrate specificity.

### 2.4. Compared to Valienamine, β-Valienamine Could Make the Tunnel of BtrR Wider and Straighter

**Figure 7:**
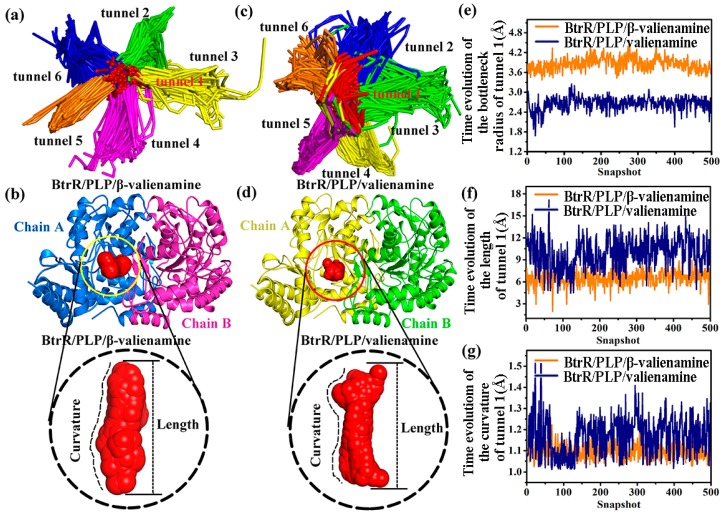
Comparison of the tunnels about the BtrR/PLP/β-valienamine and BtrR/PLP/valienamine complexes. (**a**,**c**) The top-ranked collective BtrR tunnels identified using CAVER 3.0 according to the 300-ns MD simulations trajectories. (**b**,**d**) Shape and size of tunnel 1s for BtrR/PLP/β-valienamine and BtrR/PLP/valienamine, respectively. (**e**–**g**) Time evolution of bottleneck radius, curvature, and length of tunnel 1 for BtrR/PLP/β-valienamine and BtrR/PLP/valienamine complexes, respectively.

A total of 500 snapshots were extracted from two 300-ns MD simulations for the CAVER 3.0 analysis. The structural details of the tunnel were revealed by analyzing the bottleneck residues obtained from the MD trajectory using CAVER 3.0. Table S5 showed the most frequent bottleneck residues in the tunnel: W92 (A), S187 (A), Q189 (A), K192 (A), Y342 (A), A94 (A), R221 (B), A165 (A), F336 (A), S34 (B), D219 (B), M235 (B), Y304 (A), and I93 (A). Six pathways were reliably identified using CAVER 3.0 for each simulation system (Figure 7a,c). As shown in Table 3, tunnels from the first-ranked tunnel cluster (tunnel 1) for BtrR/PLP/β-valienamine and BtrR/PLP/valienamine complexes were identified in all 500 snapshots, whereas the tunnels from the second best-ranked cluster were identified in approximately half of the analyzed snapshots. The tunnels from the remaining three clusters were rarely identified. In addition, tunnel 1 of the two complexes had a larger average bottleneck radius and smaller curvature and length than the others. This finding indicated that compared with the other tunnels, tunnel 1 was the optimal channel for the ligand to be taken off from BtrR.

**Figure 8:**
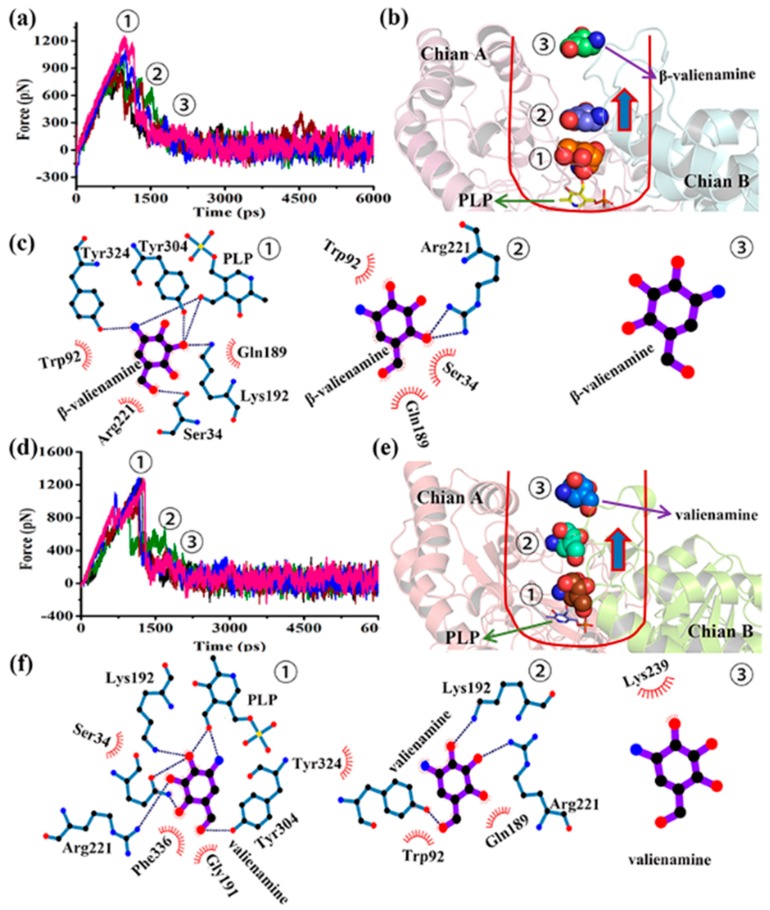
SMD simulation results. (**a**) Force analysis of valienamine dissociation from the BtrR dissociation channel. (**b**) The dynamic process of dissociation of valienamine from the BtrR dissociation channel. (**c**) Interaction between valienamine and BtrR during the SMD simulation. The dotted line represents the hydrogen bond and the curve represents the van der Waals force. (**d**) Force analysis of β-valienamine dissociation from the BtrR dissociation channel. (**e**) The dynamic process of dissociation of β-valienamine from the BtrR dissociation channel. (**f**) Interaction between β-valienamine and BtrR during the SMD simulation. The dotted line represents the hydrogen bond and the curve represents the van der Waals force.

We analyzed the changes of tunnel 1 during the two 300-ns MD simulations to explore the effects of β-valienamine and valienamine on the BtrR channels. Figure 7b and Figure 8d show the shape of two tunnel 1s for the two complexes and their relative position in BtrR. As can be seen, the shape of tunnel 1 in BtrR/PLP/β-valienamine was more regular. The time evolution of the bottleneck radius of tunnel 1 results (Figure 7e) showed that the bottleneck radius for tunnel 1 in the BtrR/PLP/β-valienamine complexes was almost 4.0 Å, which was larger than that of BtrR/PLP/valienamine (2.8 Å). The length and curvature of the tunnel 1s in the two complexes were calculated. In comparison with BtrR/PLP/valienamine, the tunnel in BtrR/PLP/β-valienamine had a smaller length and bending curvature (Figure 7f and Figure 8g). The results suggested that the existence of β-valienamine made the BtrR tunnel wider and shorter.

### 2.5. β-Valienamine Was Easier to Remove from BtrR

To examine whether the steered MD simulations could rank-order the dissociation rates of the two ligands. β-valienamine and valienamine dissociation processes were prepared. Each steered MD simulation was repeated five times. Two ligands were successfully dissociated from BtrR (Figure 8a,c). Table S6 showed the force and time needed during the stretching process. Valienamine needed more force than β-valienamine, suggesting that it needed more effort to escape from BtrR than valienamine. Furthermore, the dissociation time of the two substrates from BtrR was almost the same (Table S6). This result indicated that valienamine was more closely associated with BtrR, which was consistent with the MM-GBSA analysis in the conventional MD simulations.

To analyze the change in interaction between two different products and BtrR during dissociation, three typical conformations were selected for analysis according to the change in force during steered MD (SMD) simulation trajectories. Figure 8b,e showed the position changes of valienamine and β-valienamine in three typical conformations, which further illustrated the successful dissociation of ligands from BtrR. Figure 8c,f showed the changes of hydrogen bonds between ligands and BtrR during dissociation. More hydrogen bonds were found between the ligands and BtrR in the early stage of the SMD simulation. With the extension of SMD simulation time, the number of hydrogen bonds between ligands and BtrR decreased continuously. In Figure 8c,f, the number of hydrogen bonds between the BtrR and valienamine was significantly more than that in the β-valienamine during SMD simulations, which further indicated that β-valienamine had the advantage of leaving BtrR and contributed to the next reaction. The number of hydrogen bonds was closely related to the stability between the ligands and protein in the SMD simulation. More hydrogen bonds suggested stronger interactions with ligands, making the removal of BtrR difficult. The hydrogen bonds played an important role in the SMD simulations.

**Figure 9:**
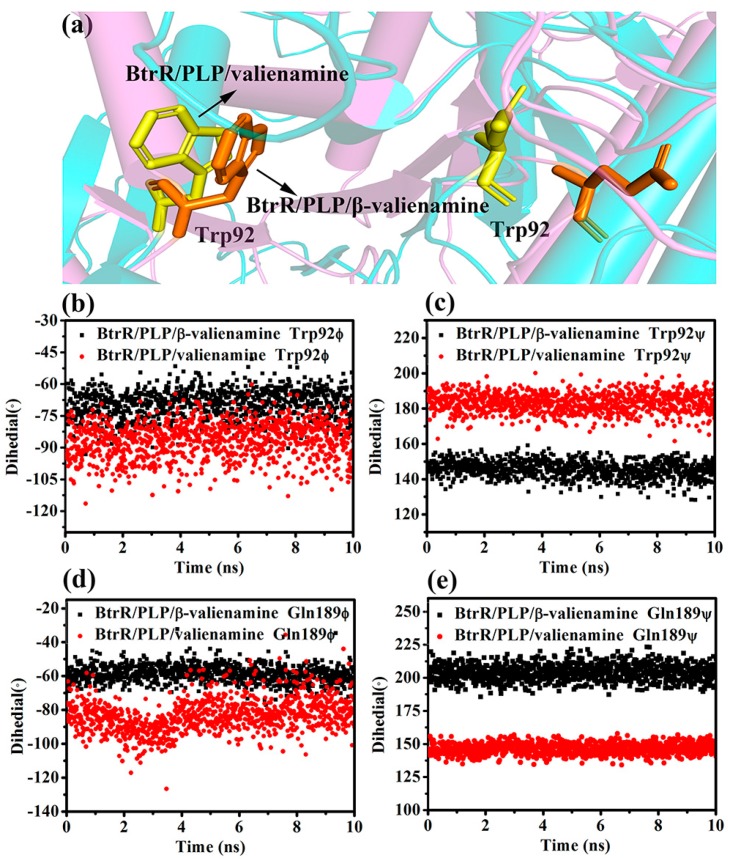
(**a**) The dihedrals of Trp92 and Gln189 in the BtrR channel. (**b**,**c**) Comparison of the dihedrals of Trp92 during the dissociation process. (**d**,**e**) Comparison of the dihedrals of Gln189 during the dissociation process.

In 2017, Vashisth et al. used potential mean force (PMF) to explore the energy changes for the departure of ligands from protein during the SMD simulations [22]. In this study, we calculated the PMF according to the second-order cumulant expansion of Jarzynski’s equality for β-valienamine and the valienamine dissociating form BtrR and the results are shown in Figure S8. As we can see, valienamine higher needed to cross a higher energy barrier (35 kcal/mol) to dissociate from the tunnel of BtrR. It indicated that valienamine had a stronger interaction with BtrR than β-valienamine, which was consistent with the results of Figure 8. Figure 8 also showed that Tyr304, Tyr324, Gln189, Lys192, Ser34, Arg221, and Trp92 played very important roles in the dissociation. The dihedral angles of Trp92 and Gln189 during the ligands dissociation were calculated and the results are shown in Figure 9. The torsion angles of two residues all have a significant difference in two SMD simulations, which may affect the distance between the side chains of two residues.

**Figure 10:**
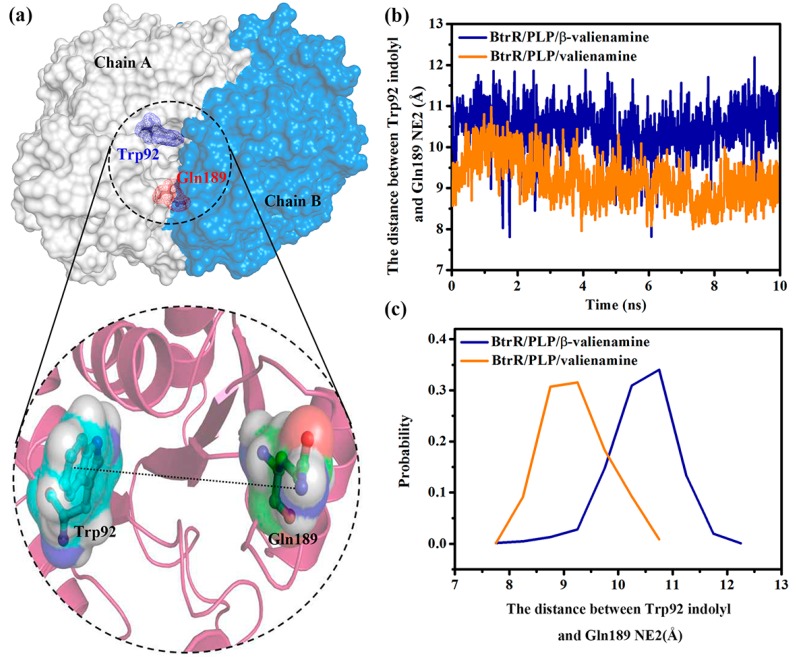
(**a**) The position of Trp92 and Gln189 in the BtrR channel. (**b**) The distance of distance between Trp92 indole group and Gln189 NE2 during the dissociation process. (**c**) Distribution probability of distance between the Trp92 indole group and Gln189 NE2.

Figure 10 shows the variation of the distance between the indole group of Trp92 and the side chain of Gln189 during dissociation. The distance between the indole group of Trp92 and the side chain of Gln189 was significantly shorter during valienamine dissociation than that found during the β-valienamine dissociation, which further indicated that Trp92 and Gln189 may provided a greater obstruction to valienamine when it was dissociating from BtrR.

## 3. Materials and Methods

### 3.1. Preparation of the Protein Structures

Two different systems were studied to identify the binding mechanism and unbind pathway of two products with BtrR: (1) homodimer PLP + β-valienamine, and (2) homodimer + PLP + valienamine. The initial coordination of the BtrR protomer was obtained from the (protein data bank) PDB bank with PDB code 5W71 [15]. BtrR exists as a homodimer that has two active sites in fairly close contact between the subunits [15]. Protonation states were determined at physiological pH by using the H^++^ server [23]. All residues were assigned in their standard protonation (pH = 7), and all missing hydrogen atoms were generated using Discovery Studio 4.0 client software [24]. The geometries of β-valienamine and valienamine were obtained from the ChemSpider database [25] and then optimized according to the density functional theory at the B3LYP/6-31G* level using Gaussian 09 software [19].

### 3.2. Molecular Docking Studies

AutoDock Vina [20], a high-precision program for virtual screening, molecular docking and drug design was used for docking. The macromolecules obtained the from PDB bank was disposed with AD4 type atoms and Kollman charges, while each heavy atom was merged with hydrogen atoms. Moreover, a grid box was added to surround all protein structures, and the grid center was located at the active sites by using AutoDock Tools. The box size of grid maps and grid-point spacing were 26 Å × 26 Å × 26 Å points and 0.375 Å, respectively. The default parameters for minimization, including pseudo-Solis, Lamarckian genetic algorithm, and Wets method, were used [26]. Each docking was repeated eight times to produce eight docking results. The conformation with the lowest energy was considered as the binding conformation for BtrR and ligands.

### 3.3. Molecular Dynamics Simulations

Two conventional MD simulations were performed using NAMD version 2.10b [27] with CHARMM27 all-atom force field parameters [28]. The homodimer bound to PLP, and β-valienamine and valienamine obtained from docking studies, were solvated in a cubic periodic box with a 10.0 Å periodic boundary condition to the closest protein atom. The remaining space in the box was filled with TIP3P water [29]. To neutralize the two systems, counter ions (Na^+^, Cl^−^) were assigned with a concentration of 0.15 mol/L. When periodic boundary conditions were present, the particle mesh ewald (PME) [30] method was carried out to deal with electrostatic interactions in each system. In our MD simulations, temperature control was performed with Langevin dynamics [31] with a 1 ps^−1^ damping coefficient (gamma), and the constant pressure control was used with all counting interactions involving hydrogen for all hydrogen atoms. To fix and release molecules in each system, minimization and equilibration were performed through a 50,000 step steepest descent algorithm before the actual MD simulations. After minimizing the systems, a (Parrinello–Rahman pressure coupling with constant particle number, pressure, and temperature) NPT (isotheral–isobaric) simulation was carried out via weak coupling to constant pressure bath (coupling time = 2.0 ps, P_0_ = 1 bar). The shake algorithm was used to constrain the bonds for the hydrogen atoms. Long-range electrostatic interactions were calculated through a PME summation algorithm [30]. The two 300-ns MD simulations were conducted at 300 K temperature and 1 bar pressure for the binding of the BtrR homodimer to β-valienamine and valienamine.

### 3.4. Pathways Identified with CAVER 3.0

The geometry-based analysis of pathways was conducted using CAVER 3.0 software according to the MD simulation trajectories [32]. It was supposed that the transport pathways with individual detailed characteristics and their time evolution to be able to confirm the pathways’ invisible form a static structure, and the pathway gating mechanism according to the structural basis was investigated. The graph made up of Voronoi edges and vertices confirmed the pathways using CAVER 3.0 [32]. In this study, 500 snapshots were extracted from the MD trajectories for CAVER 3.0 analysis to determine the tunnel where the ligand dissociated from BtrR.

### 3.5. Steered Molecular Dynamics Simulation and PMF Constraction

To explore the unbinding pathway involved in the dissociation of two products escaped from BtrR, NAMD [27] software with CHARMM27 all-atom force field [28] was used to set the ligands center mass according to the predefined direction. The initial structures extracted from conventional MD simulations were placed into a rectangular box whose size was enough to allow the simulation of tension to proceed in a direction defined by two points. The center between the C_α_ atoms of N189 and K192 (CAVER analysis showed that N189 and K192 had a higher probability to appear in the tunnel (Table S5); in addition, the two residues appeared at the edge of the tunnel) was the first point located at the active site, whereas another point was the center of mass of the ligands (β-valienamine and valienamine). The box was filled with TIP3P water molecules accompanied by counter ions (Na^+^, Cl^−^) to maintain the physiological ion conditions and neutralize the system. Subsequently, energy minimization (50,000 steps) and NPT (500 ps) were conducted. Before the SMD simulation, the velocity and direction of pulling, and force spring constant were determined. Forces of 250, 500, and 750 kJ·mol^−1^·nm^−2^ and pulling velocities of 0.15, 0.24, and 0.3 nm·ns^−1^ were used to optimize the pulling process. For the optimized parameters, the inflection point of the curve between the velocity and the maximum force required was found. The velocity below this point was acceptable and reasonable, which could accurately predict the SMD simulation results. Optimized parameters were used for the steered MD simulations. Trajectory and steering force were recorded every 1 ps. The temperature was maintained using a Nose–Hoover thermostat [33], and the pressure was controlled using a Parrinello–Rahman barostat [34]. The cut off for the van der Waals interaction was 10.0 Å. Constant-velocity ensemble simulations were carried out in our SMD simulations. The constant of the spring was set at 0.5 kal·mol^−1^·A^−2^ to pull out the imaginary atom from the accessible SMD simulation. Each system underwant a 10-ns SMD simulation, and each simulation was repeated five times.

To quantitatively visualize the variation in potential energy of the two products, we calculated the change in PMF along the egress pathway mapped out from the SMD. In those stages, a single trajectory was selected from simulations where the energy value was closest to the Jarzynski average (JA) [35,36]. The swarm of trajectories were contracted into one single JA structure, which could help remove trajectories that contributed least to the overall PMF. Our study divided the reaction coordinates into numerous small windows (≈0.2 nm), and each of them was simulated for 4 ns. Finally, after completion of all the separated simulations belonging to the same reaction coordinate, we implemented an extra simulation to recombine the output obtained from the small windows into a single PMF.

### 3.6. The Free Energy of Binding Calculation and Per-Residue Energy Decomposition Analysis

The binding free energy of BtrR bound to β-valienamine and valienamine was obtained using the molecular mechanics generalized born surface area and molecular mechanics Poisson–Boltzmann surface area approach [37,38,39,40,41]. For each MD-simulated complex, the 1000 snapshots extracted from the MD trajectory (snapshots were evenly selected from the last 2000 ps stable trajectory) to calculate the ΔG_bind_ values, and the average ΔG_bind_ value was considered as the final ΔG_bind_ value for these snapshots.

## 4. Conclusions

BtrR catalyzed valienone to β-valienamine and maintained the optical purity of the product up to >99.9%. To analyze the mechanism responsible for the high selectivity of BtrR to β-valienamine, multiple MD simulations were carried out for BtrR with two products, β-valienamine and valienamine. MD results showed that β-valienamine could enhance the hydrogen bond interaction between BtrR and coenzyme PLP by influencing the structure of BtrR Cys164-Ser167 residues. The results of energy decomposition also confirmed that the existence of β-valienamine could decrease the binding between PLP and BtrR and contribute to the catalytic reaction. CAVER analysis suggested that β-valienamine could increase the bottleneck radius, and reduce the curvature and length of the BtrR tunnel, which was conducive to the entry and removal of ligands. SMD simulation results showed that β-valienamine was more easily removed from the BtrR tunnel, thus contributing to the next catalytic reaction. Hence, from the point of view of the occurrence of the catalytic reaction or product removal, BtrR was more likely to catalyze the formation of β-valienamine.

## Figures and Tables

**Table 1:** E_gap_, IP, EA energies between the β-valienamine and valienamine.

Energy Components (eV)	β-Valienamine	Valienamine
Ionization potential (IP)	6.420	6.059
Electron affinity (EA)	0.386	0.158
Energy gap (E_gap_)	5.901	6.034

**Table 2:** Hydrogen bond occupancies between BtrR and PLP for BtrR/PLP/β-valienamine and BtrR/PLP/valienamine during MD simulations.

Hydrogen Bonds		BtrR/PLP/β-Valienamine	BtrR/PLP/Valienamine
Donor	Accepter		
Gly66:N (Chain A)	PLP:O5 (Chain A)	100.00%	100.00%
PLP:O5 (Chain A)	Glu198:OE1 (Chain A)	100.00%	68.54%
PLP:O3 (Chain A)	Gln166:OE1 (Chain A)	89.62%	0
Ser187:OG (Chain A)	PLP:O5 (Chain A)	89.11%	58.05%
Ser187:OG (Chain A)	PLP:O2 (Chain A)	87.59%	66.85%
Ser67:N (Chain A)	PLP:O4 (Chain A)	85.32%	0
Ser65:O (Chain A)	PLP:O4 (Chain A)	69.37%	26.22%
PLP:C15 (Chain A)	Asp163:OD1 (Chain A)	68.99%	20.60%
PLP:O3 (Chain A)	Gln166:CD (Chain A)	65.95%	0
PLP:O5 (Chain A)	Glu198:CD (Chain A)	88.01%	52.78%
Ser67:OG (Chain A)	PLP:C11 (Chain A)	48.10%	36.33%
PLP:O5 (Chain A)	Gly199:O (Chain A)	46.20%	0
PLP:C15 (Chain A)	Asp163:OD2 (Chain A)	44.18%	31.27%
Gly66:C (Chain A)	PLP:O2 (Chain A)	43.16%	0
Gly66:C (Chain A)	PLP:O5 (Chain A)	41.39%	37.45%
Gln189:CG (Chain A)	PLP:O7 (Chain A)	45.13%	27.34%
Ser67:N (Chain A)	PLP:O2 (Chain A)	45.88%	26.33%

**Table 3:** The top-ranked tunnels of bottleneck residues of BtrR identified using CAVER 3.0 according to the MD simulations trajectory.

Rank	Pathway Cluster	No. of Snapshots	Average Bottleneck Radius	Maximum Bottleneck Radius	Average Throughput
BtrR/PLP/β-valienamine					
1	Tunnel 1	500	3.826	4.41	0.9399
2	Tunnel 2	474	2.450	3.45	0.8375
3	Tunnel 3	350	2.235	3.13	0.8604
4	Tunnel 4	328	2.254	3.43	0.8205
5	Tunnel 5	202	1.481	2.94	0.7030
6	Tunnel 6	98	1.194	1.94	0.5484
BtrR/PLP/valienamine					
1	Tunnel 1	500	2.658	3.25	0.8967
2	Tunnel 2	432	2.134	2.99	0.8243
3	Tunnel 3	335	2.078	2.84	0.7800
4	Tunnel 4	301	1.478	2.13	0.7251
5	Tunnel 5	211	1.366	1.90	0.5374
6	Tunnel 6	108	0.927	1.03	0.6229

## Supplementary Materials

Supplementary materials can be found at https://www.mdpi.com/1422-0067/20/5/1188/s1.
