# Supplementary material for: Exploration of Catalytic Selectivity for Aminotransferase (BtrR) Based on Multiple Molecular Dynamics Simulations
Source: Int J Mol Sci. 2019 Mar 8;20(5):1188. doi: 10.3390/ijms20051188 (PMC6429434; doi:10.3390/ijms20051188)
Supplement: Supplementary file 1 [file ijms-20-01188-s001.pdf]

Figure S1. Reaction Mechanism of Aminotransferase.

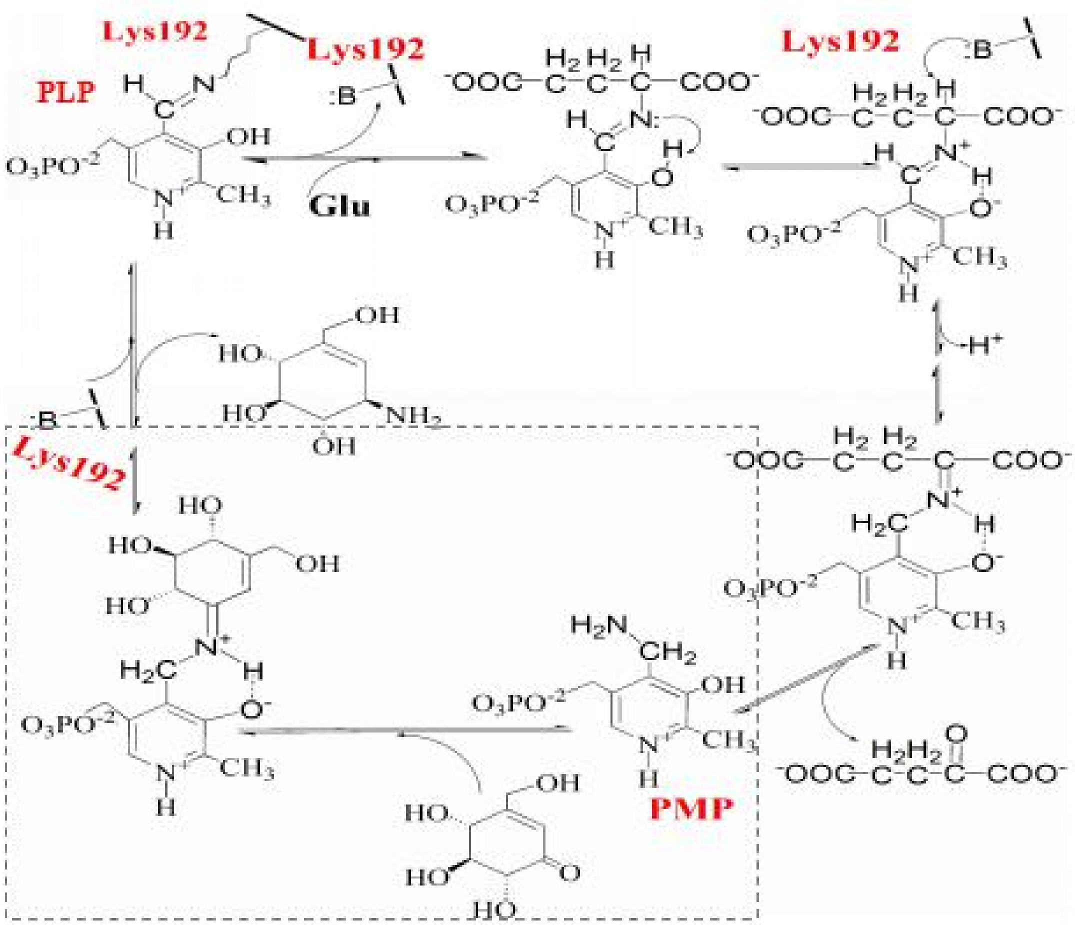

Figure S2. Comparison of the position of PLP for docking results and crystal(PDB ID: 5W71). (a) The position of PLP (cyan sticks) docking to BtrR using Autodock Vina. The PLP in crystal was shown as yellow sticks and the PLP. the residues surround the PLP were highlighted by white sticks. (b) The position of PLP (orange sticks) docking to BtrR using Autodock 4.2. The PLP in crystal was shown as yellow sticks and the PLP. the residues surround the PLP were highlighted by white sticks.

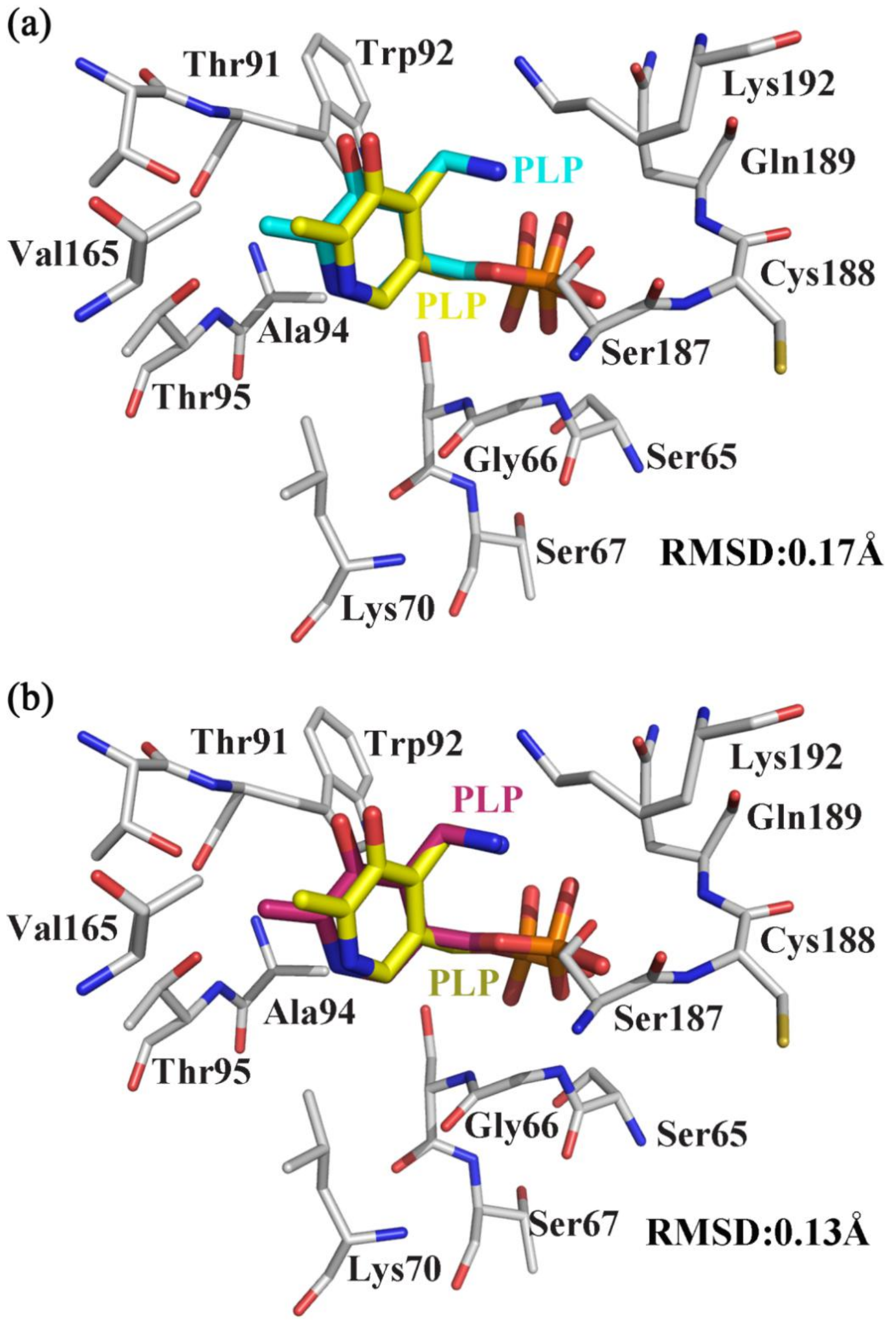

Figure S3. The relative position between Lys192 and β-valienamine/ valienamine when they were docked to the BtrR.

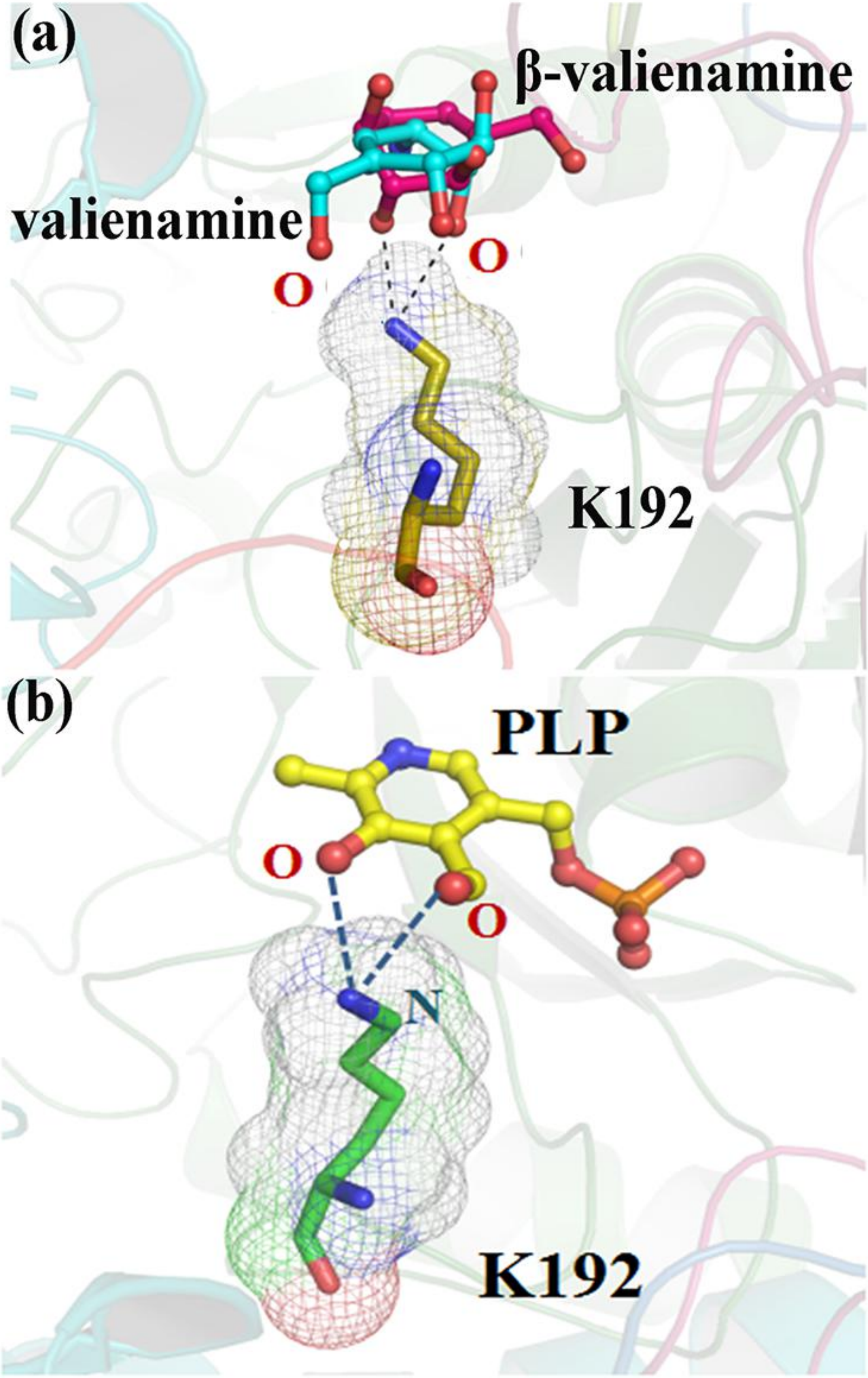

Figure S4. Cross-correlation matrix maps for BtrR/PLP/valienamine and BtrR/PLP/β-valienamine. The positive regions, marked in cyan, indicate the strongly correlated motions of residues, whereas the negative regions, colored by pink, were associated with the anti-correlated movements.

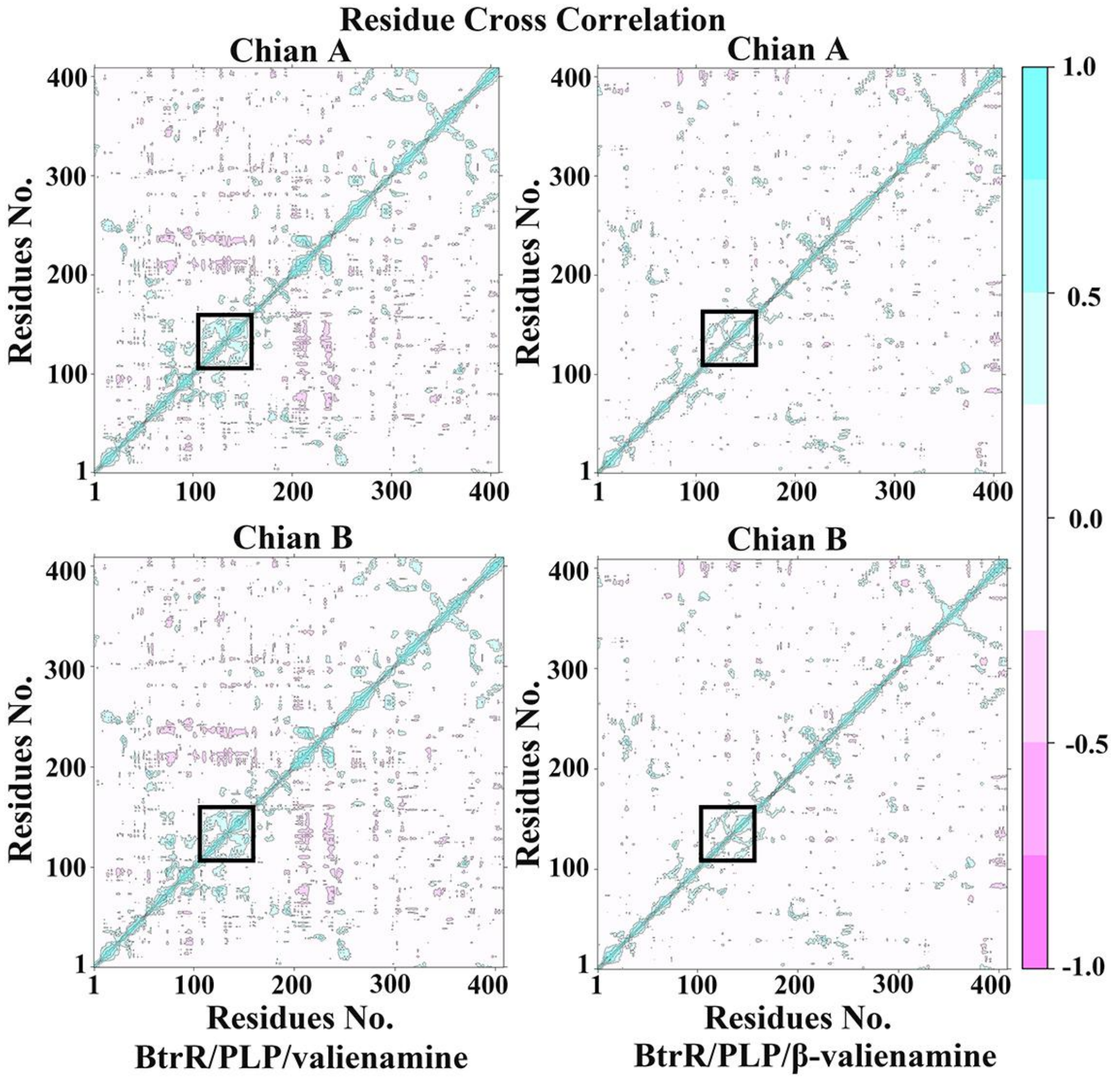

Figure S5. The distance between PLP and active pocket of BtrR**.** (a) The distance from Lys192:NZ to PLP:O6 in two complexes during the 300 ns simulations. (b) The distance from Glu198:OE2 to PLP:O5 in two complexes during the 300 ns simulations.

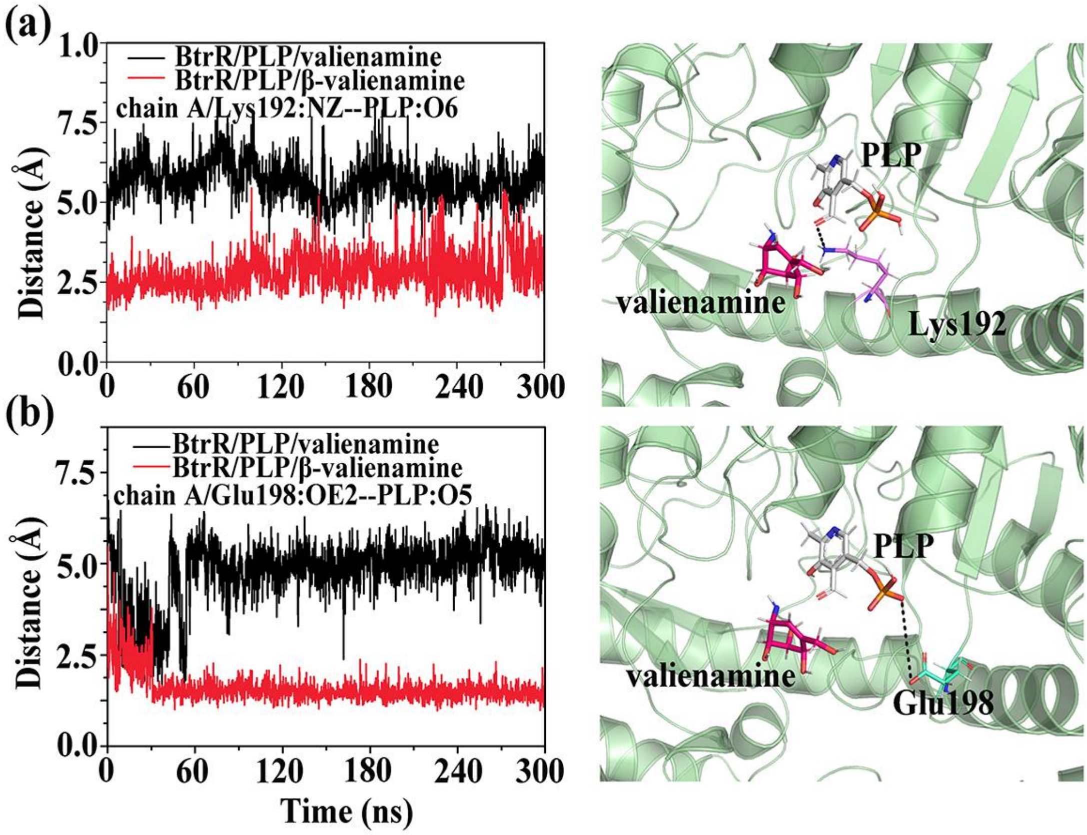

Figure S6. The distance between β-valienamine/ valienamine and active pocket of BtrR**.** (a) The distance from Gly191:N to valienamine:O1 in two complexes during the 300 ns simulations. (b) The distance from Lys192:NZ to PLP: valienamine:O2 in two complexes during the 300 ns simulations. (c) The distance from Tyr304:OH to valienamine:O4 in two complexes during the 300 ns simulations.

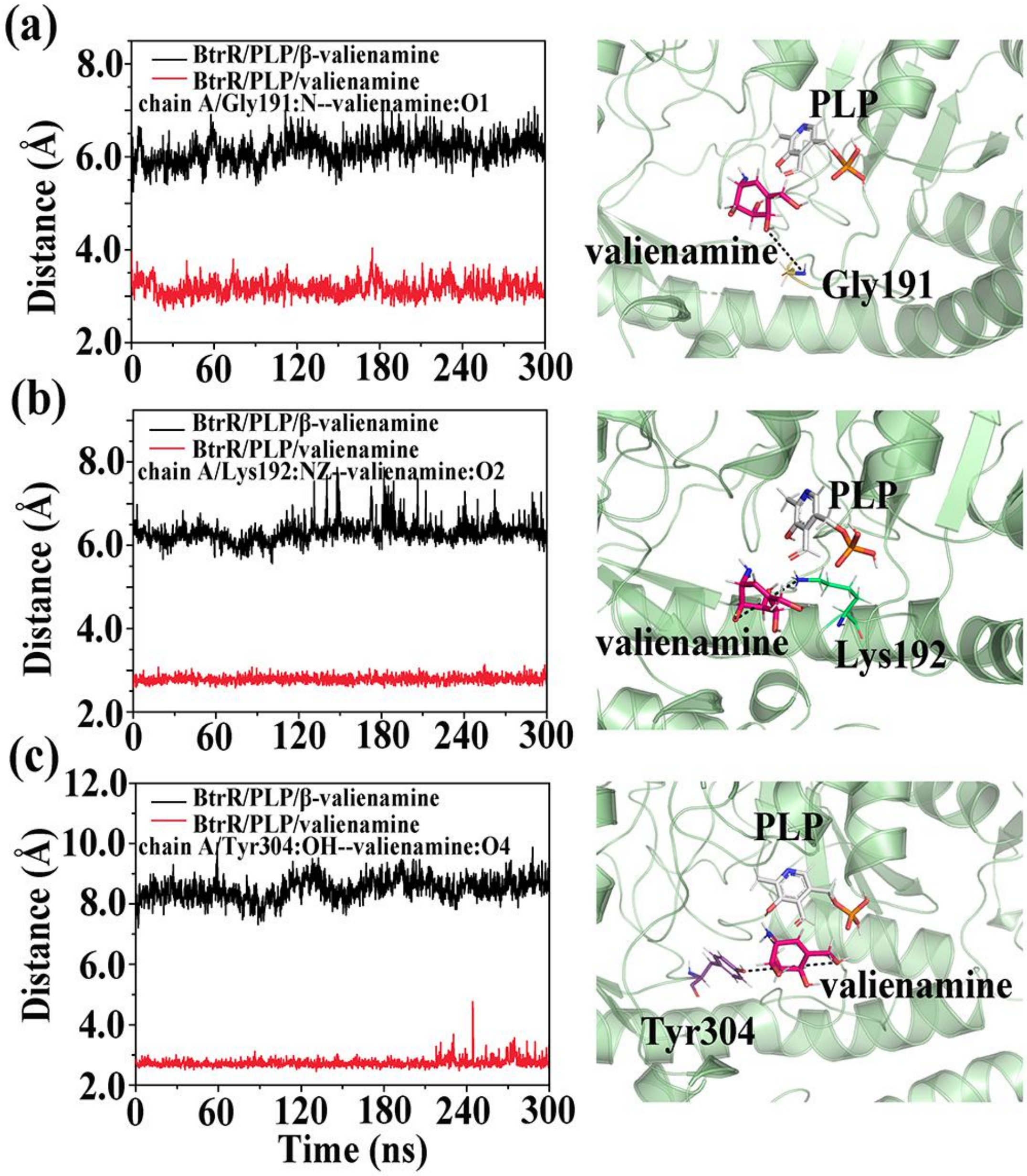

Figure S7. The relative position between PLP and the residues which had great contribution for the PLP binding to BtrR (PDB ID:5W71).

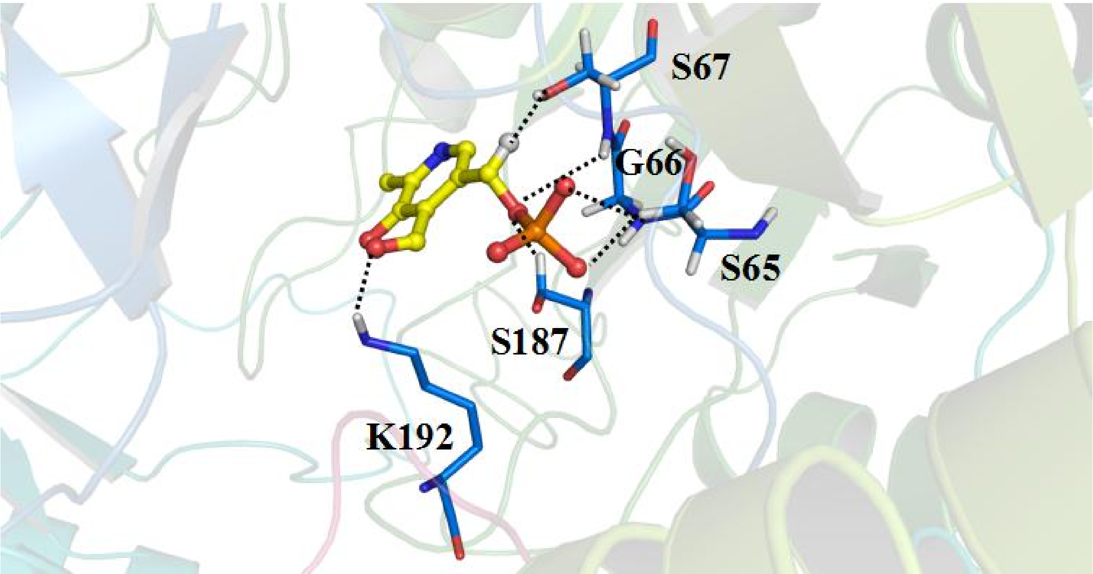

Figure S8. PMF profiles along the reactions coordinate for BtrR/PLP/valienamine (red) and BtrR/PLP/β-valienamine (blue).

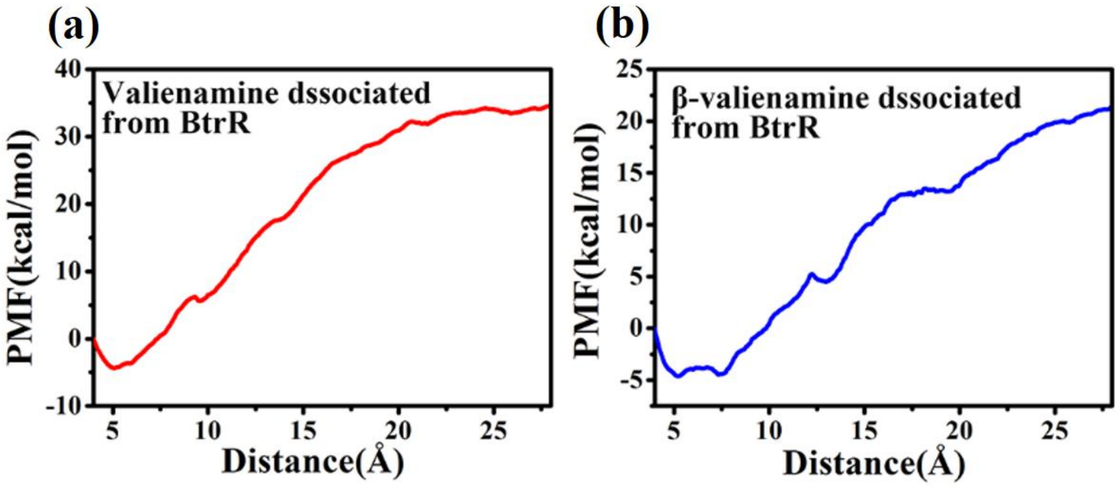

Table S1. Hydrogen bond occupancies between BtrR and β-valienamine/valienamine for BtrR/PLP/β-valienamine and BtrR/PLP/valienamine during MD simulations.

**Hydrogen Bonds**		**BtrR/PLP/**	**BtrR/PLP/**
**Donor**	**Accepter**	**β-valienamine**	**valienamine**
Gly191:N（Chain A）	valienamine:O1（Chain A）	45.9%	83.4%
Lys192:NZ（Chain A）	valienamine:O2（Chain A）	28.7%	76.9%
Ser187:NH（Chain A）	valienamine:O2（Chain A）	35.7%	56.6%
Tyr304:OH（Chain A）	valienamine:O4（Chain A）	0	46.6%
Ala165:OG（Chain A）	valienamine:O1（Chain A）	23.18%	54.32%
valienamine: N（Chain A）	Ala165:O1（Chain A）	0%	31.4%
valienamine: N（Chain A）	Gly191:OG（Chain A）	21.63%	58.97%
Tyr304: NH（Chain A）	valienamine:O4（Chain A）	35.87%	46.14%

Table S2 MM-GBSA results (kcal/mol).

	BtrR/PLP/ β-valienamine		BtrR/PLP/ valienamine	
	BtrR/β-valienamine	BtrR/PLP	BtrR/ valienamine	BtrR/PLP
ΔE_{ele}	-178.5662	-348.6766	-209.6590	-377.2256
ΔE_{vdw}	-40.2137	-48.5608	-35.9681	-62.6399
ΔG_{np}	-4.6533	-11.9789	-3.2008	-11.1948
ΔG_{pb}	140.3477	265.4528	151.3042	328.5588
$\Delta E_{ele} + \Delta E_{vdw}$	-218.7799	-397.2374	-245.6271	-439.8655
$T\Delta S$	135.6944	253.4739	148.1034	317.2640
ΔG_{bind}	-83.0855	-143.7635	-97.5237	-122.5015

Table S3 The effect of reactants on the binding energies of BtrR and PLP (kcal/mol).

	**BtrR/PLP**	**BtrR/PLP/ valienone**
**ΔE_{ele}**	-414.8499	-443.5780
**ΔE_{vdw}**	-72.3580	-91.9764
**ΔG_{np}**	-16.6164	-16.9728
**ΔG_{pb}**	367.3066	1635.5419
**$\Delta E_{ele} + \Delta E_{vdw}$**	-487.2079	-535.5544
**$T\Delta S$**	350.6902	391.2779
**ΔG_{bind}**	-136.5177	-144.2765

Table S4 Cation-π interactions in two BtrR .

	BtrR/PLP/ β-valienamine		BtrR/PLP/ valienamine	
**Cation-π**	**E(es)(kcal/mol)**	**E(vdw)(kcal/mol)**	**E(es)(kcal/mol)**	**E(vdw)(kcal/mol)**
R176(A)-F54(A)	-2.83	-2.11	-1.86	-1.74
R45(A)-Y223(A)	-4.56	-2.56	-3.27	-1.54
R272(A)-W11(A)	-2.87	-0.83	-2.83	-0.79
K192(A)-Y304(A)	-2.86	-1.48	-1.71	-0.91
K356(A)-Y359(A)	-3.34	-1.35	-3.30	-1.33
K365(A)-Y369(A)	-4.67	-0.14	-4.62	-0.12
R176(B)-F54(B)	-2.94	-2.32	-2.96	-2.28
R45(B)-Y223(B)	-4.37	-2.25	-4.30	-2.21
R272(B)-W11(B)	-2.77	-1.12	-2.74	-1.09
K192(B)-Y304(B)	-2.21	-0.72	-2.19	-0.71
K356(B)-Y359(B)	-3.21	-1.17	-3.23	-1.14
K365(B)-Y369(B)	-4.53	-0.08	-4.43	-0.07

Table S5 Probability of tunnel's residues for BtrR.

Chain	Residue	Proportion
A	W92	80.6%
A	S187	80.6%
A	Q189	80.6%
A	K192	80.6%
A	Y342	80.6%
A	A94	80.4%
B	R221	80.4%
A	A165	78.0%
A	F336	76.6%
B	S34	75.6%
B	D219	75.5%
B	M235	74.7%
A	Y304	73.5%
A	I93	73.3%
A	G66	69.9%
A	G191	69.9%
A	Q166	68.5%
A	T91	67.7%
A	D163	67.3%
A	H339	66.7%
A	T95	63.3%
A	E198	60.1%
A	T35	59.3%
A	H389	55.3%
B	K239	53.9%
B	L237	57.3%

B	V238	51.5%
A	L338	49.5%
A	S67	48.3%
A	Y337	47.7%
A	V137	43.3%
A	L70	42.7%
A	P340	40.9%
A	L350	38.3%
B	D234	39.5%
B	N247	33.5%
A	F140	33.1%
B	G35	25.7%
B	Q236	23.6%
B	C188	22.2%

Table S6. The largest applied force of ligand leaving BtrR for five parallel SMD.

	Substrates	Applied force (pN)	Time taken to dissociate from active pocket
1st	β-valienamine	1120	1322 ps
	valienamine	1300	1187 ps
2nd	β-valienamine	1127	1443 ps
	valienamine	1315	1235 ps
3rh	β-valienamine	1142	1217 ps
	valienamine	1353	1225 ps
4th	β-valienamine	1138	1343 ps
	valienamine	1375	1270 ps
5th	β-valienamine	1141	1356 ps
	valienamine	1362	1297 ps
